# Managing Persistent Hypoxemia: what is new?

**DOI:** 10.12688/f1000research.11760.1

**Published:** 2017-11-13

**Authors:** Jesús Villar, Carlos Ferrando, Robert M Kacmarek

**Affiliations:** 1 Keenan Research Center for Biomedical Science at the Li Ka Shing Knowledge Institute, St. Michael’s Hospital, Toronto, Canada; 2 Multidisciplinary Organ Dysfunction Evaluation Research Network, Research Unit, Hospital Universitario Dr. Negrin, Las Palmas de Gran Canaria, Spain; 3 CIBER de Enfermedades Respiratorias, Instituto de Salud Carlos III, Madrid, Spain; 4 Department of Anesthesiology, Hospital Clínico Universitario de Valencia, Valencia, Spain; 5 Department of Respiratory Care, Massachusetts General Hospital, Boston, Massuchusetts, USA; 6 Department of Anesthesiology, Harvard Medical School, Boston, Massuchusetts, USA

**Keywords:** mechanical ventilation, severe acute respiratory failure, hypoxemia, persistent hypoxemia

## Abstract

Mechanical ventilation is the standard life-support technique for patients with severe acute respiratory failure. However, some patients develop persistent and refractory hypoxemia because their lungs are so severely damaged that they are unable to respond to the application of high inspired oxygen concentration and high levels of positive end-expiratory pressure. In this article, we review current knowledge on managing persistent hypoxemia in patients with injured lungs.

## Introduction and context

Acute hypoxemic respiratory failure due to acute respiratory distress syndrome (ARDS) is one of the most severe forms of acute lung injury. Caused by direct (pulmonary) or indirect (systemic) insults to the lungs, it is characterized clinically by hypoxemia that does not respond to the administration of high inspiratory concentrations of oxygen (FiO _2_) and by the presence of bilateral pulmonary infiltrates on chest imaging due to high-permeability pulmonary edema ^1^. There is no specific pharmacologic treatment for ARDS. An integral part of the supportive therapy of patients with ARDS is the application of invasive mechanical ventilation (MV). The goal of MV is to achieve adequate gas exchange and tissue oxygenation without further damaging the lungs. Since the first description of ARDS ^2^, the use of positive end-expiratory pressure (PEEP) has been adopted as standard practice for the ventilator management of acute respiratory failure. PEEP prevents end-expiratory alveolar collapse.

Most patients with ARDS improve their oxygenation—as assessed by the arterial partial pressure of oxygen/FiO _2_ (PaO _2_/FiO _2_) ratio—after 24 hours of routine intensive care management and after the application of moderate to high levels of PEEP. Today, refractory hypoxemia (which, in most reports, has been defined as having a PaO _2_ of less than 60 mm Hg on a FiO _2_ of 0.8–1.0 and PEEP of more than 10 cm H _2_O for more than 12–24 hours) is an infrequent cause of death ^3^. There are no data that link a particular baseline PaO _2_/FiO _2_ to predictable structural changes in the alveolar-capillary membrane at the time of ARDS diagnosis. However, there is recent evidence showing a correlation between lung injury severity and outcome when the PaO _2_/FiO _2_ ratio is assessed under standard ventilatory settings at 24 hours of ARDS onset ^4^. Therefore, in this context, although there is no standard definition for persistent hypoxemia in terms of a predetermined PaO _2_ value under a specific FiO _2_ and PEEP for a specific period of time, for the purpose of this review persistent hypoxemia exists when the PaO _2_/FiO _2_ is not more than 200 mm Hg after 24 hours of MV. The aim of this review is to summarize the current knowledge on a number of techniques that have been shown to improve oxygenation and outcome in ARDS patients with persistent hypoxemia.

## Muscle paralysis during lung-protective ventilation

There is unequivocal evidence that MV can cause or aggravate lung damage—a concept termed ventilator-induced lung injury (VILI). Many of the pathophysiological consequences of VILI resemble those of ARDS ^5^. Since the publication of the landmark paper by the ARDS Network (ARDSnet) in 2000 ^6^ and the pooled data in a meta-analysis of six randomized controlled trials (RCTs) comparing different strategies to apply PEEP ^7^, current recommendations for ventilating patients with ARDS include the application of low tidal volumes (VTs) (4–8 mL/kg predicted body weight, or PBW), PEEP levels that maintain a positive end-expiratory transpulmonary pressure, limiting plateau pressure to less than 30 cm H _2_O, limiting driving pressure (plateau pressure minus PEEP) to less than 15 cm H _2_O, and limiting FiO _2_ to maintain a PaO _2_ of 55 to 80 mm Hg or a peripheral capillary oxygen saturation (SpO _2_) of 90% to 95%. ****These five elements are the main components of the framework for “lung-protective ventilation”.

However, despite the use of volume- and pressure-limited ventilatory strategies, mechanically ventilated patients with ARDS can be exposed to tidal hyperinflation during spontaneous inspiratory and expiratory efforts, especially in the early stages of ARDS. VT set by clinicians does not always correspond to the true VT delivered, because of double triggering, reverse triggering, and pendelluft, which can occur despite the use of analgesics and sedatives. Papazian *et al*. ^8^ examined the hypothesis that removing spontaneous respiratory efforts in ARDS patients with persistent hypoxemia would improve lung mechanics and decrease oxygen consumption. The authors performed an RCT—the ARDS et Curarisation Systematique (ACURASYS) study—in 340 ARDS patients with a PaO _2_/FiO _2_ of less than 150, a PEEP of at least 5 cm H _2_O, and VT between 6 and 8 mL/kg PBW enrolled within the first 48 hours of ARDS onset. Patients were randomly assigned to receive either a neuromuscular blockade (NMB) agent (cisatracurium) or placebo for 48 hours. The group of patients receiving muscle paralysis had lower adjusted 90-day mortality (primary outcome) and higher ventilator-free days (VFDs) at 28 days than the placebo group. The prevalence of neuromuscular weakness did not differ between groups. It is well known that NMB minimizes work of breathing and patient-ventilator asynchronies in patients with ARDS ^9^. However, the results of the ACURASYS study, seven years after its publication, remain controversial. The major criticisms of this trial include a lack of measurement of ventilator asynchrony in the control group, the Kaplan-Meier survival curves separated only after day 14, and, most importantly, the primary end-point of the trial, adjustment of 90-day mortality, achieved statistical significance only with acuity adjustment ^10^. In a recent publication ^11^, the same group of investigators examined the effects of NMB on transpulmonary pressure in a small pilot RCT of 24 patients with persistent ARDS and found that NMB could exert beneficial effects in patients with moderate ARDS by limiting expiratory efforts. Although these early data are supportive of the use of NMB, additional verification of early NMB in ARDS is required if widespread implementation is to occur. A new RCT is currently enrolling patients with moderate to severe ARDS and is powered for validating and assessing the efficacy and safety of early NMB in reducing morbidity and 90-day mortality ^12^. This trial is not an exact replication of ACURASYS since both groups of patients will receive a high PEEP open-lung ventilation approach. If the trial yields a positive result, it will establish early NMB as a standard approach in the management of patients with moderate to severe ARDS.

## Prone ventilation

ARDS is a histopathologically heterogeneous disease process ^13^. Recruitability of alveolar space with PEEP is also heterogeneous both between patients and within the lungs. Changes in posture can have profound effects on the pulmonary function of critically ill patients. Therapeutic alteration in the distribution of delivered gas for mitigating VILI is the basis of both prone ventilation and recruitment maneuvers (RMs). Prone positioning should be viewed as an adjunctive therapy to be used in combination with other accepted therapies in the management of critically ill patients with persistent hypoxemia. However, although it is widely known to improve oxygenation in patients with ARDS and shown to aid in alveolar recruitment, controversy over its use in clinical practice continues. Ventilating an ARDS patient in a prone position provides several physiological advantages for the management of persistent hypoxemia, including an increase in functional residual capacity, a change in regional diaphragm motion, better matching of ventilation to perfusion, removal of the heart’s weight from the lung, and improved secretion clearance ^14^. In general, prone ventilation can be performed safely if health-care staff are appropriately trained. Although there are sufficient data to conclude that oxygenation frequently improves when patients with ARDS are turned prone, several studies on prone ventilation produced conflicting results about its efficacy in persistent hypoxemia, until a meta-analysis suggested benefits specifically in the most hypoxemic patients receiving lung-protective MV ^15^. As with NMB, there is only one large positive RCT demonstrating survival benefit ^16^ of prone ventilation in moderate to severe ARDS, the “Proning Severe ARDS Patients” (PROSEVA) trial ^17^. The investigators randomly assigned 466 patients with persistent ARDS (as defined by a PaO _2_/FiO _2_ of less than 150 mm Hg with FiO _2_ of less than 0.6 and PEEP of at least 5 cm H _2_O) to undergo prone-positioning sessions of at least 16 hours or to be left in the supine position. In both groups, patients were ventilated using the low PEEP-FiO _2_ table from the ARDSnet trial ^6^. The 28-day mortality rates were 32.8% in the supine group and 16.0% in the prone group ( *P* <0.001), a difference that persisted at 90 days after random assignment (41.0% in the supine group versus 23.6% in the prone group, *P* <0.001).

Proponents of prone ventilation (which usually also requires NMB) suggest that the approach taken in PROSEVA was a refinement of a technique that finally got it right when patients were ventilated with a VT of not more than 8 mL/kg PBW ^18^. Detractors suggest that the large treatment effect seen (almost an absolute 20% difference) was too good to be true ^16, 19^. Of note, patients assigned to the supine position were ventilated during the first three days with very low PEEP levels (mean of 9 ± 3 cm H _2_O) for patients with severe ARDS. An additional, large validation RCT is required to confirm these findings if widespread implementation of prone ventilation in early stages of persistent ARDS is to occur. However, such a trial should ensure that the control arm receives a high PEEP open-lung ventilation approach.

## Driving pressure

Recently, attention regarding VILI has focused on driving pressure (plateau pressure minus PEEP). Amato *et al*. ^20^, in an analysis of nine pre-existing RCTs, determined that driving pressure had a greater impact on mortality in persistent ARDS than VT, plateau pressure, or PEEP. They identified a cut-point of 15 cm H _2_O. That is, the risk of death increased as driving pressure exceeded 15 cm H _2_O. Subsequently, Villar *et al*. ^21^, in a re-analysis of data from three epidemiologic studies in ARDS where all patients were ventilated with a lung-protective strategy, determined that driving pressure and plateau pressure had essentially the same impact on mortality with a driving pressure cut-point of 18 cm H _2_O. In addition, Chiumello *et al*. ^22^ identified a strong correlation between airway driving pressure and transpulmonary driving pressure (calculated as end-inspiratory transpulmonary pressure minus end-expiratory transpulmonary pressure). It seems physiologically sound to be concerned with driving pressure. The exact cut-point is still open to debate but all would agree that the lower the driving pressure the better the patient outcome.

## FiO _2_

Oxygen is routinely administered to almost all critically ill patients. Although oxygen therapy can be lifesaving, it is not without serious effects. Too little oxygen is problematic but so is too much ^23^. Rachmale *et al*. ^24^ assessed the effects of excessive oxygen exposure (defined as FiO _2_ of more than 0.5 despite SpO _2_ of more than 92%) in 210 mechanically ventilated ARDS patients on pulmonary outcomes. The authors found that prolonged exposure to excessive oxygen was associated with worsening lung function (worse oxygenation index and more days on MV), longer intensive care unit (ICU) stay, and longer hospital stay. In a subsequent RCT, Girardis *et al*. ^25^ randomly assigned mechanically ventilated medical/surgical patients to receive conservative oxygen therapy (target PaO _2_ of 70 to 100 mm Hg and SpO _2_ of 94% to 98%) or standard oxygen therapy (target PaO _2_ of up to 150 mm Hg and SpO _2_ of 97% to 100%). All other variables associated with care were standardized across groups. They found a significant difference in ICU mortality (11.6% conservative versus 20.2% standard), hospital and 60-day mortality, favoring conservative oxygen therapy. Thus, it is in the patients’ best interest to maintain the PaO _2_ of 55 to 80 mm Hg and SpO _2_ of 90% to 95% as defined by the ARDSnet protocol ^6^ to eliminate the effect of oxygenation status on outcome. Additional validation studies are in the process of being published.

## Recruitment maneuvers and transpulmonary pressure

Imaging studies have provided insight into the ARDS lung ^26^. Classic computed tomography (CT) has shown that some lung regions in ARDS appear radiographically to be relatively normal but that some other areas are partially collapsed and unable to participate in gas exchange. The concept of the “baby lung” has led to the understanding of potential interaction of MV settings and patient outcome and often using CT as a reference for applying personalized ventilatory management in patients with severe ARDS ^27^. Collapsed or atelectatic areas of the lung can be re-expanded by the application of brief periods of sustained high-inflation pressure followed by the application of adequate levels of PEEP to maintain the new re-aerated region open ^28^. These RMs are intended to re-open collapsed alveoli and to attenuate the injurious effects of the repetitive opening and closing of alveolar units, promoting lung protection by reducing lung stress in areas of heterogeneity. Three commonly used RMs are sighs, sustained inflations, and extended sighs ^29^. PEEP prevents lung unit collapse at end expiration. Much controversy exists over the benefits of RMs in persistent ARDS. A systematic review of 40 studies ^30^ showed that RMs increased oxygenation and improved respiratory system mechanics, but little information about the long-term effects and usefulness of these interventions was available until recently. The major differences seem to be based on the selection of PEEP post-RM that sustains the benefit of RMs.

In a pilot RCT that was performed from 2007 to 2013 in 200 ARDS patients with persistent hypoxemia and that compared the ARDSnet protocol ^6^ using low levels of PEEP with an open-lung approach—which involves RMs and a decremental PEEP trial for identifying the PEEP level associated with maximum dynamic compliance—Kacmarek *et al*. ^31^ found that the open-lung approach ventilatory strategy improved oxygenation and respiratory system mechanics without detrimental effects on 60-day mortality (33% in the ARDSnet group versus 29% in the open-lung approach), VFDs, or barotrauma. This trial supported the need for a large RCT using RMs in association with PEEP titrated by compliance of the respiratory system to test whether this approach is able to increase survival in patients with persistent ARDS. Such a trial has been finalized recently and we await its results ^32^.

A more recent approach for titrating PEEP is to optimize the end-expiratory transpulmonary pressure (PEEP minus pleural pressure). Pleural pressure, estimated via esophageal manometry, has been shown to differ considerably among patients with acute respiratory failure, indicating that lung and chest wall mechanics both contribute substantially and unpredictably to respiratory system mechanics and airway pressures measured by the ventilator ^33^. During RM and PEEP, the distending pressure delivered by the ventilator consists of two components: one to inflate the lung and one to expand the chest wall. Accordingly, RM and PEEP can be titrated safely to an optimal transpulmonary pressure target. In a small pilot RCT of 61 ARDS patients with persistent hypoxemia, in which the use of ARDSnet PEEP-FiO _2_ table was compared with an open-lung approach that included esophageal pressure–guided setting of PEEP (EPVent trial), targeting a positive end-expiratory transpulmonary pressure (PEEP minus esophageal pressure) showed that esophageal-guided PEEP was associated with improved oxygenation and, after adjusting for illness severity, improved survival ^34^. A multicenter validation trial powered (estimated sample size of 200 patients with ARDS) for patient-centered outcome (a composite outcome of mortality and VFDs at 28 days) is ongoing ^35^.

Of note, esophageal pressure–guided MV translated into higher PEEP application (18 versus 12 cm H _2_O on day 1), demonstrating that commonly used PEEP levels by clinicians are inadequate for optimal MV in patients with ARDS. In a small non-randomized interventional study in 14 critically ill, mechanically ventilated, morbidly obese patients, Pirrone *et al*. ^36^ evaluated both methods of titrating PEEP (that is, RM followed by a decremental PEEP trial versus RM followed by targeting a positive end-expiratory transpulmonary pressure) and observed that the two methods of determining optimal PEEP identified similar PEEP levels (20.7 ± 4.0 versus 21.3 ± 3.8 cm H _2_O) but that the PEEP levels set by the clinicians (11.6 ± 2.9 cm H _2_O) were associated with lower lung volumes, worse elastic properties of the lung, and lower oxygenation.

## Extracorporeal membrane oxygenation

This technique was originally applied to patients with severe acute respiratory failure in which it was impossible to provide adequate oxygenation by MV ^37^. Since MV is reliant on functional lung units for gas diffusion, it would be unable to provide respiratory support when there is no minimum amount of functional alveoli. Substituting alveolar gas exchange by extracorporeal membrane oxygenation (ECMO) or extracorporeal carbon dioxide (CO _2_) removal would allow a marked reduction of VT, respiratory rate, and FiO _2_, reducing the risk of VILI. To provide gas exchange during ECMO, a portion of the cardiac output must go through the ECMO circuit via the femoral, saphenous, or jugular veins. During ECMO, CO _2_ is removed by the extracorporeal circuit with MV maintained at low ventilatory rates, high PEEP levels, and with VT to maintain a plateau pressure below 29 cm H _2_O. In the last few years, there have been considerable advances in extracorporeal life support, and despite widespread and growing use worldwide in patients with ARDS ^38^, at present the evidence base for ECMO in ARDS is scarce, consisting of case series, observational cohorts, and only one RCT.

A recent RCT, referred to as the CESAR (Conventional ventilatory support versus Extracorporeal membrane oxygenation for Severe Adult Respiratory failure) trial, assessed the effectiveness of ECMO in 180 patients with severe ARDS ^39^. However, rather than directly assessing ECMO in refractory hypoxemia, investigators compared ECMO management at a referring center with MV management at tertiary centers. The 6-month survival rate was higher in patients at the ECMO center than in those patients managed with MV at participating centers (63% versus 47%, *P* = 0.03). Major concerns with the reported results included (i) patients allocated to MV were treated with conventional MV or with high-frequency ventilation, (ii) 30% of patients in the control group were not ventilated with a lung-protective strategy, (iii) the ECMO center did not treat patients randomly assigned to the conventional management group, (iv) no data regarding ventilation at study entry and during the MV period were presented, and (v) many patients randomly assigned to ECMO never received ECMO. A multicenter trial for severe ARDS comparing ECMO with a protocolized lung-protective MV strategy is ongoing ^40^.

There are some studies suggesting the combined use of ECMO with prone positioning in severe ARDS. Guervilly *et al*. ^41^ reported their experience in 15 patients with severe ARDS who were turned to a prone position during ECMO therapy because of at least one of the three following conditions: PaO _2_/FiO _2_ of less than 70 on maximal oxygenation, plateau pressure of more than 32 cm H _2_O, or failure to wean ECMO after at least 10 days on ECMO support. The authors found significant improvement in oxygenation and no complications related to proning. Also, Kredel *et al*. ^42^ reported their experience of positional therapy in a retrospective cohort of nine patients with severe ARDS treated with ECMO. Positioning therapy included complete prone, partially prone, and continuous lateral rotational therapy. During the first three days, the oxygenation index and lung compliance improved significantly, suggesting that positioning therapy can be performed safely in patients with ARDS treated with ECMO, providing appropriate precautions and a very experienced team.

## Implications for clinical practice

In summary, the most critical factor in managing the patient with ARDS is the initiation of lung-protective MV immediately upon intubation. In most patients with severe ARDS, a period of NMB agents with sedatives/narcotics is needed to gain stability of the cardiovascular/respiratory systems that are maximally stressed. Whether NMBs need to be administered for 48 hours in all patients is still open to debate, but some period from 8 to 48 hours seems beneficial in patients with severe ARDS. Once patients are stabilized, the lung should be recruited and PEEP set by a decremental best compliance PEEP trial or by PEEP establishing a positive end-expiratory transpulmonary pressure (both techniques resulting in the same PEEP). Once PEEP is set, VT is adjusted to 4 to 8 mL/kg PBW to maintain a driving pressure of less than 15 cm H _2_O and a plateau pressure of less than 30 cm H _2_O with ventilator rate increased to manage partial pressure of carbon dioxide in arterial blood (PaCO _2_). Finally, the FiO _2_ should be decreased to the lowest level that maintains the PaO _2_ of 55 to 80 mm Hg and the SpO _2_ of 88% to 95%. In patients in whom persistent hypoxemia persists, prone positioning should be considered, and in those in whom refractory hypoxemia persists after proning, ECMO should be considered. Many of the above steps in managing severe ARDS are still considered controversial since they are supported only by single RCTs, non-RCTs, or retrospective analysis. However, until data from ongoing studies are available, this seems to be the most beneficial and unifying approach to the management of the patient with severe ARDS and persistent hypoxemia.

## Abbreviations

ACURASYS, ARDS et Curarisation Systematique; ARDS, acute respiratory distress syndrome; ARDSnet, ARDS network; CO _2_, carbon dioxide; CT, computed tomography; ECMO, extracorporeal membrane oxygenation; FiO _2_, fraction of inspired oxygen; ICU, intensive care unit; MV, mechanical ventilation; NMB, neuromuscular blockade; PaO _2_, arterial partial pressure of oxygen; PBW, predicted body weight; PEEP, positive end-expiratory pressure; PROSEVA, Proning Severe ARDS Patients; RCT, randomized controlled trial; RM, recruitment maneuver; SpO _2_, peripheral capillary oxygen saturation; VFD, ventilator-free day; VILI, ventilator-induced lung injury; VT, tidal volume.
